# New Adjuvant Therapies for Obesity-Related Disorders Associated with Meta-Neuroinflammation

**DOI:** 10.3390/ph19050786

**Published:** 2026-05-17

**Authors:** Flaminia Coluzzi, Kevin Cornali, Maria Sole Scerpa, Annalisa Noce

**Affiliations:** 1 Department of Surgical and Medical Sciences and Translational Medicine, Sapienza University of Rome, 00189 Rome, Italy; 2 Unit Anesthesia, Intensive Care and Pain Therapy, Sant’Andrea University Hospital, 00189 Rome, Italy; mscerpa@ospedalesantandrea.it; 3 Department of Experimental Medicine, PhD School in Biochemistry and Molecular Biology, University of Rome Tor Vergata, 00133 Rome, Italy; kevin.cornali@students.uniroma2.eu; 4 Department of Systems Medicine, University of Rome Tor Vergata, 00133 Rome, Italy; 5 UOSD Nephrology and Dialysis, Policlinico Tor Vergata, 00133 Rome, Italy; 6 Department of Clinical Sciences, Catholic University Our Lady of Good Counsel, 1000 Tirana, Albania

**Keywords:** obesity, chronic pain, neuroinflammation, low back pain, osteoarthritis, oxidative stress, palmitoylethanolamide, gut microbiota, gut dysbiosis, cognitive impairment

## Abstract

Obesity is a complex, heterogeneous, chronic, and progressive disease, which correlates with an augmented risk of developing several comorbidities, including painful conditions, such as osteoarthritis. In this review, authors present for the first time the term meta-neuroinflammation for describing how the chronic, low-grade systemic inflammation, that occurs in obesity, may trigger oxidative stress and neuroinflammatory processes. Both the peripheral and the central nervous system are involved in neuroinflammation, leading to central sensitization and pain chronification, which leads to the observed increased incidence in obese patients of chronic pain syndromes, particularly osteoarthritis, low back pain, fibromyalgia, headache, and diabetic peripheral neuropathy. Possible mechanisms by which obesity may cause meta-neuroinflammation include adiposopathy, gut microbiota dysbiosis, and compromised integrity of blood–brain barrier, which could explain obesity-related depressive and neurodegenerative disorders. Preclinical data suggest the meta-neuroinflammation as a potential target of treatment in obese patients with degenerative joint disease. Based on these observations, targeted therapeutic strategies may include systemic administration of ultramicronized palmitoylethanolamide (um-PEA), well known for its neuroprotective, anti-neuroinflammatory, and analgesic actions, and comicronized PEA–rutin and hydroxytyrosol to restore intestinal eubiosis, with beneficial effects on body weight and mental disorders. Finally, Adelmidrol, as a PEA congener, could be considered for mitigating intra-articular meta-neuroinflammation in knee osteoarthritis.

## 1. Obesity and Chronic Pain

Obesity is a complex, heterogeneous, chronic, and progressive disease, which substantially affects more than 890 million (13%) adults [1]. In 2022, 2.5 billion adults worldwide were overweight and about 16% of the population were obese [2].

Worldwide, obesity is a major public health problem associated with increased morbidity and mortality for all-causes [3]. Although obesity is recognized as a high-risk condition for the development of other chronic degenerative non-communicable diseases, this pathology has been declared as a disease per se that leads to a reduction in the quality and expectancy of life [4].

### 1.1. Adiposopathy and Low-Grade Inflammation

In obesity patients, the adipocyte hypertrophy, visceral and ectopic adiposity, increased production of adipokines with anorexigenic function, such as leptin, and of a plethora of pro-inflammatory cytokines, such as tumor necrosis factor-α (TNF-α), interleukin (IL)-6, IL-1β, and resistin, which predisposes the organism to the adiposopathy (or “sick fat”) [5]. Adiposopathy is defined as a “pathologic adipose tissue (AT) anatomic/functional disturbances induced by positive caloric balance in genetically susceptible individuals that results in adverse endocrine and immune responses that may cause or worsen metabolic dysfunction” [3]. This condition predisposes the body to the chronic low-grade inflammation or meta-inflammation, which is observed in all tissues involved in energy homeostasis [6]. The low-grade inflammation is also sustained by infiltration of bone marrow-derived immune cells that signal via the production of cytokines and chemokines. Despite its low-grade nature, meta-inflammation negatively impacts remote organ function, a phenomenon that is considered causative of the complications of obesity. The visceral and ectopic fat, either in the liver, muscle or heart, can increase the risk of developing insulin resistance, type 2 diabetes mellitus (DM), and cardiovascular diseases (CVDs) [7], and finally increases the risk of all-cause mortality [8].

### 1.2. Inflammation, Oxidative Stress and Fibrosis in Obesity-Related Comorbidities

Notably, two-thirds of obesity-related mortality is attributable to CVDs. For every additional 2 years lived with obesity, the risk of cardio-vascular mortality rises significantly, by 7% [8]. CVDs are followed by type 2 DM, cancer (especially esophagus, colon, rectum, and liver), and chronic kidney disease (CKD) [9,10]. Obesity contributes to the development of CKD among 15–30% of patients, though direct and indirect mechanisms [11]. Among the direct mechanisms, the altered secretion of adipokines and the lipotoxicity lead to the accumulation of perirenal AT and of fatty acids in the renal parenchyma. The result is tubule-interstitial damage, which involves either the proximal tubular epithelial cells or endothelial cells [12]. The hemodynamic changes, such as glomerular hyperfiltration and microvascular stretching, and the hyperactivation of renin–angiotensin–aldosterone system are the other two direct mechanisms contributing to inflammation, oxidative stress, and fibrosis [13,14]. This triad is exacerbated if the patient presents type 2 DM, arterial hypertension, and atherosclerosis, namely comorbidities and indirect mechanisms of obesity itself that are responsible for the development and progression of CKD [11].

Obesity is also related to other structural and functional abnormalities that reduce patients’ quality of life (QoL). These include gastrointestinal reflux disease, gallbladder disease, osteoarthritis (OA), obstructive sleep apnea/obesity hypoventilation syndrome, psychological and eating behavior disorders, anxiety and depression, and impairment of physical performance [15,16].

Chronic pain is a widespread health issue, which affects over 20% of the adult population [17,18]. In obese patients, chronic pain syndromes are among the most common observed comorbidities, with their relevant psychosocial consequences. These two phenomena are closely related, with each condition adversely impacting the other, because of limited mobility, mood disorders, and common chemical mediators [19].

#### 1.2.1. Obesity and Osteoarthritis

Numerous studies investigated the relationship between OA-related pain and obesity, which is the focus of this review. Among older people, long-term obesity has been identified as a significant predictor of pain, particularly with an increased risk of back, hip, and knee pain [20], which are the most common sites of OA. Among OA-related pain syndromes, low back pain (LBP) has the highest prevalence in the general population, affecting over 600 million people globally. LBP is supposed to be a major determinant of age-standardized disability-adjusted life-years (DALYs) in the next 25 years, while improvement of body mass index (BMI) has been proposed as one of the strategies for improving life expectancy [21]. Overweight and obesity have been recognized as risk factors for LBP. Obesity increased the incidence of LBP, with an odd ratio of 1.36 and 1.4, respectively in men and women [22]. In a recent cohort study conducted in Norway, higher values of BMI have been associated with higher incidence of LBP, particularly among very obese women [23]; however, the exact magnitude of this phenomenon is still under investigation.

Advancing age and adiposity may contribute to musculoskeletal degenerative diseases, which lead to sarcopenic obesity (SO), a condition that links osteopenia/osteoporosis, muscle loss, and obesity [24]. SO is associated with reduced physical activity, loss of independence among older adults and is a determinant risk factor for frailty [25]. In postmenopausal women, SO had greater effect on knee OA compared to obesity without sarcopenia and to sarcopenia without obesity [26].

#### 1.2.2. Obesity and Other Chronic Pain Syndromes

The hypothesis that obesity is linked to chronic pain because of joint overload is currently overtaken by the evidence of many other pain syndromes, commonly associated with obesity, such as painful diabetic peripheral neuropathy [27], headache [28], and fibromyalgia [29].

Diabetic neuropathy is the most common complication of DM, affecting about 50% of DM patients and about 70% of patients with diabetic neuropathies receive medications for neuropathic pain [30]. Visceral obesity is an independent risk factor for diabetic peripheral neuropathy [31].

Different studies investigated obesity as a risk factor for headache/migraine. Although migraine frequency was not associated with BMI, obese patients seem to have a higher prevalence of frequent and severe headache/migraine [32,33,34,35]. The exact relationship between these two comorbidities is not fully understood; however, there is evidence that obesity could be a consequence of migraine, through the effect of inflammatory mediators, adipokines, and alterations of gut microbiota [36].

Finally, 35% of adults with fibromyalgia are obese and obesity impacts most of the clinical features of fibromyalgia, such as tenderness and stiffness, fatigue, physical functioning, sleep, and cognitive function, leading to a reduced QoL [37]. Among women suffering from fibromyalgia, obese patients displayed higher levels of anxiety and depression, compared with the normal-weight subjects [38]. Even in this case, it is not possible to understand whether obesity is a cause or a consequence of fibromyalgia [29].

Women exhibit a higher susceptibility to chronic diseases, such as osteoarthrosis and fibromyalgia. Moreover, they are more likely to be diagnosed with obesity. Therefore, we could suppose sex/gender differences in the development of meta-neuroinflammation. In particular, sex-dependent neuroinflammatory mechanisms have been identified as potential determinants of brain function and psychiatric diseases in women, where hormonal fluctuations represent the most powerful modulators of neuroimmune dynamics. Neuroinflammatory vulnerability in women arise also from enhanced microglial activity in response to cytokines, greater cortisol reactivity, and enhanced interactions between estradiol, glucocorticoid receptors, and neuroimmune signaling [39].

These gender differences should be taken into account when mechanistically driven approaches are considered for managing neuroinflammation-based chronic diseases. Obese women are more likely to be vulnerable to meta-neuroinflammation, but they could benefit from better results when treated with molecules targeting neuroinflammation, such as palmitoylethanolamide (PEA). However, currently the heterogeneity in study design, the lack of sex-stratified data and hormonal status, limit the generalizability of these hypotheses.

The main objective of this narrative review was to explore the hypothesis of meta-neuroinflammatory pathways underlying several disorders related to obesity. We suggested identifying the obesity-related disorders associated with meta-neuroinflammation—a neologism used for describing the systemic chronic low-grade inflammation—as a potential trigger of oxidative stress and neuroinflammatory processes. We highlighted the possible role of pathological AT in neuroinflammation, through the alterations of gut and blood–brain barrier (BBB) permeability and the sensitization of peripheral and central nervous system (CNS).

Additionally, we focused on potential targeted therapeutic agents for patients with obesity, namely autacoid local injury antagonist amides (ALIAmides). Among these, PEA and adelmidrol (ADM) were discussed in this review for their anti-inflammatory, analgesic, and immunomodulatory properties. Regarding PEA, micronized-PEA (m-PEA), ultramicronized PEA (um-PEA) and its co-micronized formulations with rutin and luteolin (Lut)- namely co-mPEA-rutin and hydroxytyrosol (HTyr), and co-ultraPEA-Lut, appear to act as powerful modulators of meta-neuroinflammation and oxidative stress pathways.

Moreover, these bioactive lipid compounds show therapeutic potential in regulating glucose and lipid metabolisms through the activation of peroxisome proliferator-activated receptor (PPAR)-α. PEA and ADM are increasingly recognized as valuable components of the therapeutic armamentarium for managing painful conditions, particularly OA, fibromyalgia, and neuropathic pain, which are commonly observed in obese patients.

## 2. Search Methods

To ensure a comprehensive and transparent approach, this narrative review followed a structured search strategy. An extensive literature analysis was conducted across PubMed/MEDLINE, Scopus, EMBASE, and the Cochrane Central Register of Controlled Trials (CENTRAL). The search covered the period from June 2005 to June 2025, capturing two decades of advancements in the field.

The search strategy employed Boolean operators (AND, OR) to combine keywords related to the three main pillars of the review: (1) obesity (“obesity”, “adiposopathy”, “sarcopenic obesity”); (2) pathophysiological mechanisms (“neuroinflammation”, “meta-inflammation”, “oxidative stress”, “blood–brain barrier”); and (3) therapeutic targets (“ALIAmides”, “palmitoylethanolamide”, “adelmidrol”, “nutraceuticals”).

Studies were selected according to the following inclusion criteria: (i) original research (both preclinical and clinical), (ii) systematic and narrative reviews, (iii) practice guidelines, and (iv) studies focusing on the interaction between obesity-related inflammation and chronic pain syndromes (e.g., osteoarthritis, fibromyalgia, neuropathy). The exclusion criteria were: (i) articles not published in English, (ii) case reports or small case series (*n* < 5), (iii) editorial letters or commentaries, and (iv) studies not directly relevant to meta-neuroinflammation or ALIAmides.

The quality of the included clinical studies was assessed on study design, sample size, and the relevance of outcomes to the article’s objectives. A total of 283 references was ultimately included to provide a comprehensive overview of the topic.

## 3. Mechanisms Underlying Obesity-Induced Oxidative Stress and Meta-Neuroinflammation

AT is an endocrine organ distributed throughout the body and is characterized by high metabolic and dynamic activity [40]. AT regulates several physiological mechanisms through the secretion of adipocytokines (also called adipokines) into the bloodstream, creating a communication with other tissues and organs [41]. In adult mammals, AT is classified in two different types: white and beige. White AT (WAT) accounts for the largest percentage of AT in the human body and is localized around the viscera, subcutis, and perivascular. WAT stores excess energy in the form of triglycerides and secretes adipokines and vasoactive factors. Its phenotype changes in patients affected by obesity, becoming hyperplasic and hypertrophic, suffering the infiltration of the immune cells and secreting vasoconstrictor factors. Beige AT (BAT) mainly surrounds the thoracic aorta. It possesses anti-inflammatory and cardioprotective properties and is involved in the thermogenesis, dissipating energy as heat. For this reason, BAT has anti-obesogenic and anti-diabetic properties, ensuring cardio-metabolic health [42]. Lean individuals with normo-metabolic function present an increased production of anti-inflammatory ILs (like IL-10, IL-5, IL-4, IL-13, IL-25, IL-33) and anti-inflammatory adipokines (such as adiponectin, omentin, apelin and secreted frizzled-related protein-Sfrp-5) [43]. Moreover, in a healthy AT, macrophages constitute 5–10% of the cells, of which only a small part is in a pro-inflammatory state (M1) because the remainder of the resident macrophages is in an anti-inflammatory state (M2) [44]. Patients with obesity are characterized by the meta-inflammation in which AT is mainly represented by hypertrophic adipocytes that accumulate lipid droplets, secrete pro-inflammatory adipokines (like leptin, resistin, and visfatin), and amplify the infiltration into AT itself of pro-inflammatory cells (such as M1 macrophages, T helper-TH 1 cells, natural killer-NK cells, CD8^+^ T cells, neutrophils, and mast cells) [45].

### 3.1. From Meta-Inflammation to Meta-Neuroinflammation

A growing amount of literature in the last few years is focusing on the systemic effects of adiposopathy, as a consequence of an increased production of inflammatory cytokines. Most comorbidities associated with obesity have been directly related to a chronic low-grade inflammatory state, well known as metabolic inflammation, or meta-inflammation. Metabolic dysfunction has been clearly associated with chronic pain; however, the exact mechanism leading to central sensitization has only been recently identified in the close interlink between persistent peripheral cytokine expression and neuroinflammation, which involves peripheral and CNS. Immune and metabolic challenges have been shown to induce changes in the gene networks associated with pain perception, dopaminergic synapses, and glutamate signaling pathways, in the hypothalamus [46].

On the other side, neuroinflammation is by itself a physiological reparative process, with a specific role in the evolutive phase of the CNS. In particular, activated microglia plays a key role in the regulation of neurogenesis, the promotion of neurons survival, synaptic pruning, and the phagocytosis of neuronal cells or debris that is no longer needed. In adults, neuroinflammation mainly protects the CNS against external agents; however, when chronically activated it may represent the basis of several neurological diseases and contribute to pain chronification [47] through central sensitization [48].

The evidence that obesity leads to meta-inflammation, oxidative stress, and BBB disruption strongly supports the hypothesis that neuroinflammation could explain the increased incidence in these patients of chronic pain syndromes and CNS degenerative diseases, as discussed in this review. The theory of an immune-metabolic network, as the physiopathological linking between meta-inflammation and neuroinflammatory-mediated diseases, has led authors to move from the concept of obesity and meta-inflammation to the evidence of obesity-induced meta-neuroinflammation.

### 3.2. Obesity and Neuroinflammation

The CNS requires a highly controlled microenvironment to support its physiological functioning. This is possible thanks to the presence of three biological barriers at the blood–brain interface that effectively separate the brain from the rest of the body [49]. These include the BBB, the blood–cerebrospinal fluid barrier, and the arachnoid barrier [50]. The BBB is an anatomo-functional structure that protects the CNS from systemic circulation, not allowing the pro-inflammatory factors, toxins, immune cells, and pathogens to be translocated into the brain [51].

The integrity of the BBB is compromised in patients with obesity. Therefore, the disruption of the basement membrane (BM) in the BBB allows the extravasation of leukocytes. Leukocytes express highly glycosylated molecules on their surface, namely the P-selectin glycoprotein ligand-1 (PSGL-1), consenting selectins adhesion receptors to bind them and triggering the neuroinflammation response through the activation of microglia. The interaction between PSGL-1 and P-selectin and E-selectin mediate the initial capture and the rolling of leukocytes on the vascular endothelium in search of a point for extravasation, which can occur by paracellular and transcellular diapedesis. Most transmigration into the perivascular space occurs via a paracellular mechanism. The immune cells extend pseudopods and pass through the endothelium, thanks to the interaction with platelet endothelial cell adhesion molecule (PECAM) and junctional adhesion molecule-A (JAM-A). When leucocytes cannot find an endothelial junction, transcellular diapedesis occurs [52,53]. The leukocyte extravasation into the brain parenchyma is also permitted by matrix metalloproteinase (MMP)-9, which removes away BM filaments [54]. The impaired BM also becomes thicker, leading to increased vascular permeability [55]. This process is favored by the activation of protein kinase C (PKC), advanced glycation end-products (AGEs), transforming growth factor-β (TGF-β) and connective tissue growth factor [55]. AGEs act on AGE receptor (RAGE) to intensify nuclear factor kappa β activation (NF-κβ), increasing pro-inflammatory gene expression, including RAGE itself and pro-inflammatory cytokines, like TNF-α [56].

Astrocyte endfeet wrap around the entire CNS vascular tree and perform important functions in regulating the BBB, through the cerebral blood flow, nutrient uptake, and waste elimination [57]. During meta-neuroinflammation, astrocytes produce and secrete a wide range of molecules and chemokines to attract circulating peripheral immune cells (CD8^+^ T cells, B cells, NK cells, monocytes, and macrophages) into the CNS [58]. Conversely, astrocytes can boost effector functions of peripheral immune cells through the production of IL-15. TH 17 cells promote pathogenic activities of astrocytes by expressing the receptor activator of nuclear factor-kappa β (RANK) ligand and granulocyte-macrophage colony-stimulating factor (GM-CSF). The RANK activation by TH 17 cell–expressed RANK ligand triggers the production of C-C motif chemokine ligand (CCL)-20, inducing the recruitment of effector T cells in the CNS [59]. In astrocytes, GM-CSF causes the expression of pro-inflammatory genes [60], creating a cytotoxic state with consequent BM destruction [55].

Chronic overexposure of vascular endothelial growth factor (VEGF) also increases the expression of intercellular adhesion molecule-1 (ICAM-1) and major histocompatibility complex (MHC) class I and II expression, modulating immune responses in the CNS through opening of the BBB and allowing contacts between CNS antigens and blood-borne immune mediators [61].

Activated microglia migrates to the injured area and releases proinflammatory cytokines, nitric oxide (NO), reactive oxygen species (ROS), prostaglandins, and chemokines, resulting in the additional chemoattraction of circulating leukocytes [62]. Moreover, leptin leads to the activation of the mechanistic target of rapamycin (mTOR) and hypoxia-inducible factor 1 (HIF-1) in the endothelial cells of the CNS, leading to VEGF production [63]. VEGF signaling triggers the activation PKC-β and Rho-kinase (ROCK), exacerbating neuroinflammation [64]. Activation of PKC-β is also due to increased diacylglycerol concentrations, typically observed in hyperglycemic conditions [65]. This pathway increases the activity of nicotinamide adenine dinucleotide phosphate (NADPH) oxidase, producing O_2_^−^. The latter mediates the phosphorylation of the inhibitor of kappa β kinase (IKK) and induces downstream degradation of Iκβα, leading to the nuclear localization and transcriptional activation of NF-κβ [66], disrupting the BBB [67].

ROCK-mediated cellular pathway inhibits the expression of endothelial nitric oxide synthase (NOS), which reduces the availability of NO, inducing endothelial dysfunction [68] and increased vascular stiffness [42]. Mediators of obesity-induced endothelial dysfunction also include an altered sirtuin 1 expression, oxidative stress, autophagy machinery, and endoplasmic reticulum stress [68].

Inactivation of endothelial NOS causes the activation of microglia, promoting a pro-inflammatory phenotype in the brain, downregulating the claudin-5 and occludin, and increasing the BBB permeability [69]. Moreover, endothelial NOS-deficient mice exhibit impaired cognitive performance, suggesting that loss of endothelial NO has a detrimental effect on the functions of neuronal cells [70]. At the same time, an increased NO production in the CNS is associated with the pathogenesis of neurodegenerative diseases, such as Parkinson’s disease (PD), and Alzheimer’s disease (AD) [71]. In fact, the pathological manifestations of AD include not only the accumulation of amyloidbeta-protein (Aβ) and hyperphosphorylated tau (pTau) in the brain, but also microgliosis, astrocytosis, and neurodegeneration mediated by meta-neuroinflammation [72].

Aβ also increases ROCK-1 activity in neurons [73] and, in turn, ROCK-1 enhances cleavage of the amyloid precursor protein, producing increased Aβ formation [74].

### 3.3. Obesity and Oxidative Stress

Meta-inflammation induces a reduction in endogenous antioxidants, namely superoxide dismutase (SOD), catalase, and glutathione peroxidase (GPx). In fact, the amplified production of adipokines by hypertrophic adipocytes causes an increase in ROS production, resulting in oxidative stress [75]. Enhancement levels of ROS contribute to oxide lipids and proteins, resulting in reduced SOD activity [76]. Moreover, in obese patients, the excessive caloric intake causes mitochondrial dysfunction, contributing to the formation of O_2_^−^ [77].

At the same time, hyperglycemia leads to amplification of mitochondrial oxidative phosphorylation and ROS production. The latter react with NO to generate peroxynitrite, which mediates MMPs activation and tissue inhibitor of metalloproteinases inhibition, causing BM degradation [55,78]. The BM regeneration is unable to compensate the protease activity of the MMPs. In fact, the increase in fibronectin, collagen IV, and laminin compromises the attachment of cells to the BM and the downregulation of heparin-sulfated proteoglycans removes anionic protein binding sites, destabilizing the BM [79].

**Figure 1:**
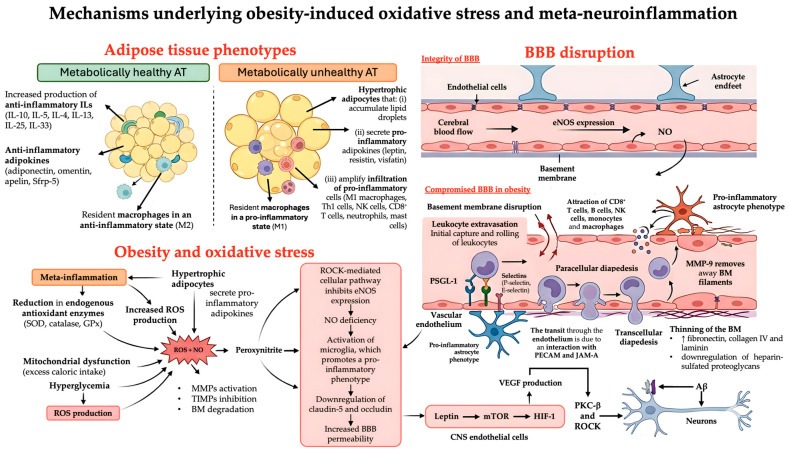
Mechanisms underlying obesity-induced oxidative stress and meta-neuroinflammation. Abbreviations: AT, adipose tissue; Aβ, beta-amyloid; BBB, blood–brain barrier; BM, basement membrane; CNS, central nervous system; eNOS, endothelial nitric oxide synthase; GPx, glutathione peroxidase; HIF-1, Hypoxia-Inducible Factor-1; ILs, interleukins; JAM-A, Junctional Adhesion Molecule-A; MMPs, matrix metalloproteinases; mTOR, mechanistic Target of Rapamycin; NK cells, Natural killer cells; NO, nitric oxide; PECAM, Platelet Endothelial Cell Adhesion Molecule; PKC-β, Protein Kinase C beta; PSGL-1, P-selectin glycoprotein ligand-1; ROCK, Rho-kinase; ROS, reactive oxygen species; Sfrp-5, secreted frizzled-related protein; SOD, superoxide dismutase; Th1 cells, T helper 1 cells; TIMPs, tissue inhibitors of metalloproteinases; VEGF, vascular endothelial growth factor.

The mechanism underlying obesity-induced oxidative stress and meta-neuroinflammation are summarized in Figure 1.

### 3.4. Sarcopenic-Obesity and Irisin Pathway

Irisin is an adipo-myokine hormone produced during physical exercise through the expression of the peroxisome proliferative activated receptor-γ coactivator-1 α (PGC-1α) [80]. Irisin binds to its integrin αV/β5 receptor with these consequences (i) WAT browning; (ii) improving of insulin sensitivity and metabolic balance, by enhancing mitochondrial functions and by reducing oxidative stress; (iii) promoting osteogenesis and mitigating the bone loss; (iv) attenuating the cognitive dysfunction, by decreasing Aβ toxicity, neuroinflammation, and oxidative stress, and by improving brain-derived neurotrophic factor (BDNF) signaling, which rescues cognition and synaptic health; (v) regulating dopamine pathways, alleviating neuropsychiatric symptoms, like depression and apathy; and (vi) mitigating cardiac injury [81,82].

The levels of irisin are significantly lower in patients with obesity, osteoporosis, sarcopenia, AD, and CVDs [82]. The dysfunctional phenotype caused by low levels of irisin is exacerbated in patients with SO. In fact, SO compromises mitochondrial oxidative capacity and lipid oxidation in skeletal muscle and suppresses sarcolipin-induced sarcoplasmic reticulum calcium ATPase (SERCA) activation, impairing the ability to switch between glucose and lipid metabolism in response to nutrients and physical exercise. Moreover, this impairment results in reduced oxidative capacity, diminished energy expenditure, and increased adiposity [83]. SO patients display a smaller total gray matter volume [84] and show higher serum levels of IL-6, IL-18, TNF-α, TNF-like weak inducer of apoptosis (TWEAK), and leptin compared to non-sarcopenic patients; in contrast, the levels of insulin growth factor 1, insulin, and adiponectin are significantly lower [85]. For these reasons, irisin may represent a therapeutic potential biomarker for metabolic diseases, osteoporosis, sarcopenia, and neurodegenerative diseases [81].

### 3.5. Osteoarthritis and Biomarkers

Current diagnostic methods detect OA only in its advanced stages, thereby limiting prevention perspectives, and patients with this condition are treated symptomatically.

In the last few years, attention has been drawn to disease-associated molecular biomarkers, which can be identified in readily accessible biofluids, such as blood, urine, and the synovial fluid and may be useful for predicting OA progression. In particular, the BIPEDS system has been developed by the Food and Drug Administration for classifying biomarkers, according to B (burden of disease), I (investigative biomarkers still to be defined), P (prognostic), E (efficacy of intervention), D (Diagnostic), and S (Safety) [86].

Biomarkers of inflammation, such as IL-6, TNF-α, and myeloperoxidase, have been identified as relevant indicators of disease activity. IL-6 and TNF-α promote joint inflammation and synovial degradation. Elevated levels of these pro-inflammatory cytokines correlate with knee radiographic OA and cartilage loss. Normalizing mast cell activities in the OA joints significantly reduces the concentration of IL-6 and TNF-α [87].

Beyond traditional inflammatory markers, other emerging biomarkers are currently under investigation, including metabolites, noncoding RNAs, and cartilage degradation markers. Interestingly, although blood-derived biomarkers are nowadays the most commonly studied, other biofluids, such as urine and synovial fluid may express increased levels of biomarkers, such as various degradation products of type II collagen [88].

Specific indicators of activated macrophages (CD14 and CD163) and neutrophils (SF elastase) have been detected in the synovial fluid of OA patients [89].

Biomarkers have been used for stratifying OA patients in different subclasses and targeting treatment according to endotypes, or disease subgroups defined by a distinct pathophysiological mechanism. Inflammatory endotype is characterized by high levels of cytokines in the synovial fluid and nociceptive pain. Metabolic endotype is associated with dysmetabolic diseases, such as obesity and DM, which support the meta-inflammation and the nerve sensitization. In these patients, leptin and adiponectin are useful biomarkers.

Bone remodeling endotype can be detected by increased markers of subchondral bone turnover, while senescent endotype, mediated by cellular aging, can be monitored by cartilage matrix degradation products [90].

In a recent systematic review, no relevant circulating biomarkers in blood, urine, synovial, and cerebrospinal fluids have been found to be associated with OA-related pain. Serum total cholesterol was the only biomarker consistently associated with pain [91].

These results indirectly support the hypothesis that inflammation of metabolic origin contributes to nerve sensitization and neuroinflammation, which mediates pain amplification and chronification.

Specific markers of neuroinflammation have been identified for targeting different glial populations. Microglia cells and astrocytes, which are the main non-neuronal key actors in the CNS, express a wide range of inflammatory mediators and several receptors. However, nowadays, these have a role only in preclinical studies, where they are used to assess the activation state of cells involved in neuroinflammatory processes [92].

## 4. Meta-Neuroinflammation and Oxidative Stress in Osteoarthritis

In the last few years, OA has been identified as a degenerative disease, sustained by an inflammatory chronic condition, where neuroinflammation and oxidative stress probably play a key role. Obesity has been recognized as a predisposing factor, due to low-grade chronic inflammation. Meta-neuroinflammation could represent a possible common pathway for both obesity and OA.

### 4.1. Osteoarthritis and Neuroinflammation

In addition to being triggered and aggravated by biomechanical trauma, OA is known to be an inflammatory chronic condition [93,94]. As for other chronic inflammatory diseases, OA-related pain is sustained by inflammatory responses in peripheral tissues, e.g., joints, as well as in the peripheral and CNS. Such phenomena are described as “neuroinflammation”, and they rely on a bidirectional signaling between nervous structures and cells and the peripheral damaged tissues, possibly as a compensatory response against the peripheral damage [95]. Animal models of OA showed that joint neurons, especially high-threshold C and Aδ afferents, undergo plastic changes [96] and develop sensitization, hence mechanical stimuli are perceived as painful in behavioral tests [97,98]. After the induction of knee OA via intra-articular monosodium iodoacetate (MIA) injection, destabilization of the medial meniscus (DMM), or partial meniscectomy, animals display mechanical hyperalgesia [99]. Nociceptors that are initially responsive to certain stimuli, namely cold, heat, or chemicals, and silent to mechanical stimuli, become mechanosensitive, with a polymodal phenotype [100]. Higher responses to the same stimuli also occur in dorsal root ganglia (DRG) neurons [101]. Here, glial cells, such as astrocytes [102] and, especially, microglia, the resident macrophages of the CNS, which are activated during neuroinflammatory processes [103,104]. Microglia activation in the DRG occurs in animal models, with different timing based on the OA inducers, ranging from one week after MIA injection, and 8 to 16 weeks after DMM [105]. Related pain behaviors, thermal allodynia, mechanical allodynia, and hyperalgesia, are related to microglia activation, as they are reversible after glial inhibition [106,107]. Microglial cells produce and release cytokines and molecules, such as TNFα, IL-1, nerve growth factor (NGF), and substance P, which may further activate similar cells in a paracrine manner, and sustain their shift to a M1 proinflammatory phenotype [108].

Under physiological conditions, chondrocytes, fibroblast-like synoviocytes, synovial macrophages, and mast cells (MCs) are present in the joints in a quiet state, as “sentinels” against pathogens and possible injuries [109,110]. Particularly, MCs play a key role in OA-induced neuroinflammation. They secrete granules containing proinflammatory substances, such as histamine, proteinases (tryptases and chymases) [111], as well as chemokines and cytokines (TNF-α, IL-1β, IL-6, IL-8, CCL2, VEGF, and others), thus leading to vasodilatation, angiogenesis, and the recruitment of other inflammatory cells from the bloodstream [112]. MCs also produce NGF [113], which binds to neurotrophin p75 and tropomyosin-related kinase (TRK)-A receptors on several inflammatory cells, including other MCs, promoting their degranulation, as well as sprouting of pain fibers [114], microglia activation in the dorsal horn [105], and other structural changes leading to neuroinflammation and pain chronification [115]. Inhibition of both NGF and TRK-A induced analgesia in animals; therefore, it was applied for pain management in OA [116,117].

When inflammation occurs, the glycation of the extracellular matrix (ECM) allows for the release of collagen-derived AGEs, which may hinder neuronal cell attachment and neurite formation, and elicit neuronal excitation, neuropeptide and neurotransmitters release, and eventually peripheral sensitization. Such effects are counteracted by morphine administration, thus suggesting that glycated extracellular matrix (ECMGC) may represent a new target for chronic pain treatment [118]. A specific trigger for ECM degradation during joint inflammation is lipid peroxidation, which is enhanced in OA synovial cells compared to healthy controls [119]. NO, released by chondrocytes in inflamed joints [120], triggers the production of lipid peroxidation products, such as 8-isoprostane F2α [121], malondialdehyde, and 4-Hydroxy-2-nonenal [119], as well as ECM degradation by MMPs [120] and inhibition of proteoglycans and collagen synthesis [122]. On the other hand, a growing body of evidence suggests a role for NO as an inhibitor of the NF-κB pathway and a stimulator of collagen synthesis in vitro [120]. ECM degradation also leads to the activation of the complement cascade [123], and the release of damage-associated molecular patterns into the joint cavity, as well as inflammatory, catabolic, and chemoattractant factors. All of these are responsible for increased BBB permeability, spinal infiltration of monocytes and their differentiation into activated microglial cells, eventually leading to the sensitization and excitation of DRG neurons [124] and cerebral areas associated with pain perception, such as the thalamus. Similarly, cytokines and chemokines, namely prokineticin (PK)-1 and PK-2, were upregulated in brain areas that are typically related to mood control, such as the prefrontal cortex and the hippocampus, therefore explaining mood disorders, particularly anxiety and depression, associated with MIA-induced OA in mice, alongside allodynia, motor deficits, and fatigue. Interestingly, OA symptom burden seems to be more severe in women than in men in clinical practice [125,126], possibly due to higher inflammatory responses [127,128] and suggesting sex-related peculiarities in the crosstalk among peripheral damaged tissue and the CNS. Also, in animal models, older subjects display higher levels of inflammatory markers in the DRG and the spinal cord, and in peripheral tissues, counteracted by morphine administration [129].

### 4.2. Osteoarthritis, Oxidative Stress and Mitochondrial Dysfunction

Oxidative stress has been linked to various inflammatory and degenerative conditions [130], and may be a contributing factor for OA pathogenesis and progression [131,132], as demonstrated both in vitro and in vivo [133]. An imbalance between the production of ROS and antioxidant defensive mechanisms occurs, with negative impact on joints and pain development [134]. Overexpression of anti-inflammatory molecules, namely sestrin2 (Sesn2) [135], and the inhibition of pro-inflammatory ones, such as glycogen synthase kinase-3β (GSK-3β) [136,137], reduced ROS and cytokines levels in the spinal cord, in MIA- and complete Freund’s adjuvant (CFA)-induced OA, respectively, with analgesic effects. OA-related pain is often undertreated, and the inhibition of oxidative processes may be a new target for treatment [138]. Mitochondria are key players in the oxidation pathways. Damaged mitochondria are continuously replaced by new ones, in order to preserve mitochondrial function: this process is known as mitochondrial biogenesis. Mitochondria are highly dynamic entities [139], with damaged ones getting removed through autophagy [140]. When such mechanisms are impaired, a reduction in ATP generation occurs, alongside augmented ROS production, mitochondrial DNA mutations, and mitochondrial membrane dysfunction [141], all linked to a wide range of degenerative and inflammatory diseases, including onset and progression of OA [142], as assessed in animal models [143] and human chondrocytes [144].

Several transcription factors regulate mitochondrial renewal, namely PGC-1α [145], nuclear factor erythroid 2-related factor (Nrf)-1 and Nrf-2, and mitochondrial transcription factor A [146].

Nrf-2 positively modulates the expression of many endogenous antioxidants, namely NAD(P)H oxidoreductase 1 (NQO1), heme oxygenase-1 (HO-1), SOD, glutathione (GSH), and GPx [147,148], and inhibits pro-inflammatory pathways, such as NF-κB [149], hence reducing levels of inflammatory cytokines [150]. Nrf-2 also promotes macrophage differentiation to a M2 anti-inflammatory phenotype [151], modulates osteoclastogenesis [152], and inhibits the activation of inflammatory synovial fibroblasts [153], which are responsible for synovial and ECM degradation through the production of degrading factors, such as MMPs and disintegrin and metalloproteinase with thrombospondin motifs (ADAMTS) [154,155]. Nrf2 induction in models of surgically induced OA prevented OA progression via inhibition of NLR family pyrin domain containing 3 (NLRP3) inflammasome [156], a complex with a pivotal role in triggering inflammatory responses in several pathological conditions [157]. NLRP3 inflammasome is upregulated in the synovial tissue of mice with collagen-induced arthritis [158], as well as in sensory neurons in the DRG in MIA-induced OA. Accordingly, its inhibition prevented pain chronicization in such models [159].

Concentrations of N-acetylaspartate, a marker for neuronal integrity produced in oligodendrocytic and neuronal mitochondria, were reduced compared to healthy controls, but returned to normal-range levels after total knee arthroplasty, thus suggesting that surgical treatment may counteract the maladaptive mechanisms leading to pain sensitization and chronification in subjects with OA, possibly via ameliorated mitochondrial function [133].

GSH is a well-known antioxidant with pleiotropic effects, including the activation of Nrf2 [160]. GSH and its precursor molecule, N-acetylcysteine, have a role in resistance to oxidative stress [161], reducing inflammation markers and cartilage degradation, as well as better pain control and functionality [162]. A way to increase GSH levels is through hydrogen sulfide: administration of slow-releasing hydrogen sulfide donors, which are known to boost GHS, alleviated mechanical allodynia, grip strength, and memory deficits, and depressive-like behaviors accompanying OA through inhibition of activated microglia and downregulation of inflammation makers, namely inducible NOS, all while maintaining high levels of antioxidant/detoxicant molecules in central regions such as the hippocampus, the amygdala, periaqueductal gray matter, and infralimbic cortex [163,164,165].

## 5. Therapeutic Perspectives

In patients suffering from obesity, targeted treatment of disorders associated with meta-neuroinflammation using novel adjuvant therapies, in combination with lifestyle modifications and first-line pharmacological treatment, could represent an innovative approach to the management of chronic pain syndromes, as well as adiposopathy, gut microbiota dysbiosis, and neuroinflammation.

The modification of dietary habits and regular physical exercise, combined with traditional pharmacological interventions, can have a significant positive impact on the central nervous system–gut–AT axis [166,167,168]. The role of the Mediterranean diet (MedDiet) in improving metabolic health, alleviating meta-inflammation, and modulating neuroinflammation processes is well established [169]. The MedDiet is rich in fiber, polyunsaturated fatty acids (PUFAs), bioactive phytochemicals [170], and essential micronutrients, all of which contribute to its recognized benefits for both metabolic and neurological health. Moreover, the MedDiet plays a pivotal role in modulating host metabolism and in profoundly influencing the composition and function of the gut microbiota and organs with a metabolic function. Similarly, therapeutic ketogenic regimens offer neuroprotective benefits through anti-inflammatory and metabolic optimization mechanisms [171].

Physical activity can also modulate gut microbiota composition and optimize gut–brain axis signaling pathways, potentially mitigating neuroinflammation through its systemic metabolic effects [172].

Alongside lifestyle modifications, first-line pharmacological therapies are recommended due to their superior efficacy in promoting weight loss and improving cardiovascular and metabolic health in patients suffering from obesity [173].

The pleiotropic effects of glucagon-like peptide-1 receptor agonists (GLP-1RAs), already approved by the Food and Drug Administration for the treatment of type 2 DM and obesity [174], could be repurposed for the treatment of neuroinflammation and neurodegenerative pathways [175,176,177]. GLP-1RAs have been associated with anti-inflammatory, neurotrophic, and neuroprotective properties in preclinical models of neurodegenerative disorders, operating through a dual mechanism of action [178]. In fact, GLP-1RAs can mitigate insulin resistance and suppress the complex neuroinflammatory cascade [179]. There is growing evidence to suggest that GLP-1RAs are a promising and readily available therapeutic strategy for disrupting the core inflammatory and altered metabolic pathways that are common in many neurodegenerative conditions [180]. However, further research through human clinical trials, characterized by long-term follow-up, is required to confirm the safety, tolerability, and efficacy of GLP-1RAs in reducing neuroinflammation, and to clarify their potential in the treatment of neurodegenerative diseases [179].

Based on the preclinical and clinical evidence reported in the scientific literature, we propose a “think outside the box” approach that includes the use of nutraceuticals and dietary supplements belonging to the ALIAmide family, such as PEA and ADM. These molecules possess anti-inflammatory, analgesic, and immunomodulatory properties [181]. NAEs are therefore considered powerful modulators of meta-neuroinflammation and oxidative stress [182]. These bioactive lipid compounds have potential therapeutic perspectives for controlling glucose and lipid metabolism [183,184,185].

The activation of PPAR-α, directly by PEA and indirectly by ADM, may control hepatic lipid homeostasis by stimulating fatty acid oxidation and adapting the metabolic response to lipid overload and storage [186,187].

The close relationship between dysfunctional AT and innate immune cells in the brain (mainly microglia) and in periphery (mast cells) could be an intriguing target of intervention. PEA and ADM are becoming increasingly relevant molecules with a growing reality in the therapeutic armamentarium for managing painful conditions, particularly for OA, fibromyalgia, and neuropathic pain, which are commonly observed in obese patients [181,188,189,190].

### 5.1. Palmitoylethanolamide

PEA (N-hexadecanoylethanolamide) is a N-acylethanolamine (NAE), with anti-inflammatory and analgesic properties [191]. Due to its high lipophilicity, when orally administrated, PEA is only able to cross the BBB in small amounts [192]. Micronization and ultra-micronization are used to reduce its size and improve its uptake through the gastrointestinal tract [193,194].

After absorption, PEA is hydrolyzed to ethanolamine and palmitic acid by fatty acid amide hydrolase (FAAH), which is a membrane-bound enzyme with a heterodimeric structure, located in the endoplasmic reticulum and acting on multiple substrates, including N-acylamines, NAEs, and N-acyltaurines [195]. PEA is also metabolized by FAAH-2, which is localized in lipid droplets [195], and by lysosomal enzyme NAE acid amidase [196]. Such enzymes act on either endogenous [197,198,199] or exogenous [200,201] PEA, and are found in different tissues and cytotypes, ranging from the gastrointestinal tract [199,202], to joints [203], to the brain [204,205,206].

#### 5.1.1. Preclinical Data

In the CNS, PEA is produced, released, and hydrolyzed by microglia [192]. Its involvement in neuroinflammatory processes has been thoroughly studied, and it may be mediated by its direct and indirect interaction with various receptors, in particular the PPAR-α [192], which is itself involved in several pathways [207], including proinflammatory responses [208].

The modulation of neuroinflammation by PEA is mediated by non-neuronal cells of the nervous system, particularly mast cells (both in the peripheral and CNS) and microglia (at spinal and supraspinal levels), through a close interaction with neurons located centrally, spinally, or peripherally [192].

The preclinical effects of PEA on neuroinflammation associated with neurodegenerative and other neurological disorders have been investigated in animal models of both PD and AD.

In PD mouse models, the PPAR-α receptor-mediated effects of m-PEA and of co-ultraPEA-Lut have been shown to reduce neuroinflammation by decreasing the expression of inducible NOS and cyclooxygenase (COX)-2 [209,210], as well as IL-1β and TNF-α [211].

In animal models of AD, chronic treatment with um-PEA reduced neuroinflammation and oxidative stress thorough PPAR-α receptor-mediated mechanisms, including the inhibition of IL-6 increase in the hippocampus, the reduction in inducible NOS and caspase-3 activation and the decrease in ROS production [212,213]. Additionally, chronic administration of co-ultraPEA-Lut prevented the upregulation of COX-2, IL-1β, and TNF-α gene expression, while restoring IL-10 mRNA levels [214].

Moreover, the um-PEA directly counteracts the inflammation and the mitochondrial dysfunction in a PPAR-α-dependent manner [215].

Epidemiological studies indicate that AD and PD risk positively correlate with metabolic diseases such as DM and metabolic syndrome (MetS) [216].

Meta-inflammation and neuroinflammation are closely interlinked, creating a vicious cycle between obesity and brain dysfunction. Saturated fatty acids and pro-inflammatory cytokines associated with obesity-induced meta-inflammation act as primary mediators, transmitting signals from dysfunctional AT to the brain, particularly to the hypothalamus [217]. The temporal course of meta-neuroinflammation following obesity onset, particularly at the hypothalamic level, can be conceptualized as a three-phase process: Initiation Spark phase (first 24 h), the Adaptive Transition phase (days to weeks), and the Dysfunctional Phase. The early phase is characterized by rapid increases in pro-inflammatory cytokines and glial activation. This is followed by transient gliosis, BBB alterations, and compensatory neuronal and structural responses, whereas the chronic phase involves persistent gliosis, reprogramming of neuroinflammatory signaling, structural deterioration, and progressive functional decline [218].

Obesity-driven neuroinflammation has been associated with disruption of the BBB in the hippocampus [219]. In an experimental model of high-fat diet (HFD)-induced obesity, um-PEA limited albumin extravasation and restored tight junction gene expression [215].

PEA has been extensively studied for its pleiotropic effects at both central and peripheral levels. Its ability to limit obesity-related disorders associated with meta-neuroinflammation has been demonstrated in an experimental model of HFD-induced obesity. um-PEA appears to modulate the inflammatory response by inhibiting the NF-κB signaling pathway in the hypothalamus, with a consequent reduction in pro-inflammatory cytokines such as IL-1β. In the hippocampus, it decreases TNF-α and IL-1β levels, and in the periphery, it reduces MCP-1 and LPS [215].

In addition to controlling meta-inflammation and neuroinflammation, PEA’s neuroprotective effects also lead to improvements in neurotransmitter imbalances associated with behavioral dysfunction [215].

The time-dependent effects of m-PEA formulations in modulating meta-neuroinflammation have been partially characterized in preclinical studies. The effects of PEA on restoration of inflammatory homeostasis can occur within days to weeks after treatment initiation, rather than leading to a rapid and complete resolution of inflammation. In animal models of chronic inflammation, sustained administration with m-PEA or um-PEA is required to achieve a more pronounced attenuation of meta-neuroinflammation [215,220,221,222,223].

Hence, PEA has potential benefits in several pathological and inflammatory conditions. Its ability to revert astrogliosis [224,225] and learning and memory impairments in mice [213], makes it a promising molecule for management of human neurodegenerative disorders [214,226].

In addition, PEA has an “entourage effect” on other receptors and pathways, such as cannabinoid receptors (CB)-1 and CB2 [227], as well as non-CB1 and non-CB2 [228], and transient receptor potential vanilloid 1 (TRPV1) channels [229] with paracrine activation of other microglial cells [230], as well as MCs [231]. Administration of exogenous PEA was found to be effective against MC-mediated acute and neurogenic inflammation [232], to reduce neuropathic pain [233], and to improve functionality and pain control in small animals with chronic OA [234].

PEA is present in high concentrations in healthy joints and acts as a MCs modulator [231]. Physiologically, MCs, alongside macrophages, represent about 3% of resident cellular cell population and act as sentinels for possible pathogens and injuries. In arthritic joints, the number of MCs increases via maturation and proliferation of resident cells, as well as recruitment of progenitors from the blood stream via paracrine mechanisms [112]. PEA reduces the degranulation of MCs in vitro [235] and in vivo [236], and was found to reverse histopathological changes in OA rat modals, alongside a reduction in joint swelling, levels of proinflammatory markers, and cartilage-degrading MMPs [237].

PEA may be beneficial in metabolic dysfunctions [238], since it was found to promote white-to-beige AT conversion [186] and reduce fat mass, especially when combined with other antioxidant and anti-inflammatory compounds, such as rutin [239].

In HFD-fed mice, the long-term administration of PEA (30 mg/kg/die per os) limited hepatic lipid accumulation, increased energy expenditure, and reduced insulin resistance. Moreover, mechanistic studies indicated that the effects of PEA on lipid metabolism are attenuated by AMP-activated protein kinase (AMPK) inhibition. All these findings identify PEA as a modulator of hepatic lipid and glucose homeostasis, limiting metabolic inflexibility induced by nutrient overload [183].

PEA may also have modulating effects in intestinal inflammatory conditions [240,241]. The bidirectional communication between the gastrointestinal tract and the CNS, defined as “gut microbiota–brain axis” [242], occurs through the neuroendocrine [243], autonomic and enteric nervous systems [244], with the activation of the immune system and the production of bacterial metabolites by the gut microbiota [245]. On the other hand, the gut microbiota exerts a significant influence on both human physical and mental health [246]. Gut dysbiosis may be a consequence of HFDs and Western diet [247], drugs, immune system dysfunction, and stress itself [248]. Particularly, gut dysbiosis is associated with a reduction in short-chain fatty acids (SCFAs), which are normally produced by bacterial fermentation in the gastrointestinal tract [249], and increase tight junctions protein expression both in the intestinal epithelial barrier [244] and the BBB [250]. A reduction in SCFAs occurs in gut dysbiosis, with translocation of bacterial lipopolysaccharide [245,247] through disrupted gut barrier into the bloodstream [251], hence triggering systemic inflammation [252], further hypothalamic–pituitary–adrenal axis activation [253], and eventually paving the way for chronic-metabolic diseases [254], such as type 2 DM, obesity, CKD, arterial hypertension, inflammatory bowel disease, as well as autoimmune, neoplastic, neurodegenerative disorders, and even chronic pain [255,256,257,258,259,260]. In chronic pain patients, gut microbiota may be affected by opioid administration [261] and drugs used for managing opioid induced constipation [262]. Restoring the eubiosis of gut microbiota could be beneficial to nervous peripheral and central disorders related to gut dysbiosis [263]. PEA has been shown to reduce the permeability of the human gastrointestinal tract in vitro, ex vivo, and in vivo [264], and to reduce gut inflammatory response and gut dysbiosis in HFD-fed mice, through a limitation in immune cell recruitment and activation of intestinal MCs and macrophages [223].

#### 5.1.2. Clinical Data

As already suggested by preclinical studies, PEA has been investigated as add-on therapy for migraine [265] or for neurodegenerative disorders [266], as well as against adiposopathy. Regarding metabolic disorders, the co-mPEA–rutin and HTyr were proved as a potential conservative treatment for MetS patients. It has been shown that an eight-week treatment with co-mPEA–rutin and HTyr, combined with a tailored calorie-controlled MedDiet, significantly reduced the body weight, BMI, fat mass, and inflammation biomarkers (like C-reactive protein and erythrocyte sedimentation rate), compared to placebo-supplemented patients. At the same time, the fat-free mass, phase angle, and body cell mass were increased [267]. These preliminary results, obtained from 19 patients with MetS, are currently being confirmed by the authors in a larger study population, in order to evaluate the role of co-mPEA–rutin and HTyr also in the regulation of glucose and lipid metabolism.

Currently, PEA supplementation at a dose of 700 mg per day for 12 weeks has been shown to significantly reduce serum triglyceride levels in 58 overweight adults [184].

In the field of pain management, m-PEA administration was useful against neuropathic features in patients with LBP. Although m-PEA may not have a role on functional improvement when administrated alone at low doses (600 mg/die) [268], um-PEA showed promising results at a higher dosage (600 mg twice a day) as a support therapy during rehabilitation, with improvement in mental and physical components of QoL, and disability scores [269]. Add-on therapy with um-PEA was correlated with reduced intake of opioids in patients with chronic LBP, with significant reduction in pain perception and neuropathic manifestations, overall maintaining a good tolerability and safety profile [270,271], which makes um-PEA a good option even in older patients [272]. In addition, um-PEA ameliorated pain control in patients with failed back surgery syndrome as an add-on therapy, together with dual opioid tapentadol and anticonvulsant pregabalin [273].

It is well known that neuroinflammation plays a key role in the onset and evolution of chronic pain and probably also in its transition from acute to chronic phase. Supplementation with oral m-PEA formulations for the management of chronic pain appears to have time-dependent effects. As well as the advantage of early treatment, extending treatment beyond the first month has an overall beneficial effect, especially for those patients with incomplete pain management [274].

With regard to gut dysbiosis, only a randomized, placebo-controlled, double-blind study conducted by Batacan and co-authors investigated the role of PEA on the gut microbiome of overweight adults (BMI 30–40 kg/m^2^), at the dose of 700 mg/day for 12 weeks, with PEA reducing triglycerides and IL-2 levels. No significant differences were observed in overall microbiota composition after PEA administration; the microbiota richness and diversity remained constant for both groups [184].

In light of these results, PEA seems to be a promising agent for both obesity and chronic pain conditions as an add-on therapy; however, current clinical evidence is still weak, and further studies are needed to establish its clinical role in meta-neuroinflammatory processes. Further investigations are also warranted to better determine the action of PEA on gut dysbiosis in patients suffering from obesity [275,276].

PEA has most often been used by adults in doses of 300–1200 mg by mouth daily. No indications are available regarding the optimal dosage in obese patients. Clinical trials have always been conducted at a standard dosage, without dosing adjustments based on body weight. However, it is reasonable to believe that obese patients may require higher doses, because lipophilic drugs are usually administered according to the total body weight.

### 5.2. Adelmidrol

Being PEA insoluble in water, another member of the ALIAmides family, named ADM, a synthetic derivate of azelaic acid, has been investigated as particularly suitable for topical and intra-articular administration, because of its both amphipathic and amphiphilic properties [277]. Data are currently available for its use in mouse model of colitis [278], acute lung injury [279], and other skin and mucosal inflammatory conditions [280,281].

#### 5.2.1. Preclinical Data

The protective effects of ADM on non-alcoholic steatohepatitis (NASH) were evaluated at three different doses (5, 10, and 20 mg/kg/die) in an HFD-induced mouse model, with intraperitoneal administration once daily for 7 weeks. ADM-treated mice showed a significant reduction in hepatic transaminase levels (aspartate aminotransferase and alanine aminotransferase), along with a dose-dependent improvement in liver histopathology compared to the control group. ADM administration also resulted in decreased levels of TNF-α, triglycerides, and total cholesterol, as well as increased levels of adiponectin, high-density lipoprotein cholesterol, and hepatic GSH. Moreover, a marked decline in MMP-1 levels was observed [185]. These findings suggest that ADM exerts its protective effects by modulating inflammatory pathways and enhancing metabolic processes, possibly through the activation of different receptors such as PPAR-α, PPAR-γ, and CB2. Overall, this study highlights the potential of ADM as protective agent in liver diseases associated with metabolic disorders but further research, aimed at exploring its efficacy and its mechanisms in humans, are necessary [185].

A combination of 2% ADM with 1% high molecular weight hyaluronic acid (HA) has been approved for intra-articular injections in knee OA, with a significant improvement in analgesia and functionality [277]. In OA joints, ADM acts as a PEA enhancer, leading to higher PEA levels [282]. Moreover, ADM leads to a reduction in inflammatory cytokines and cartilage degradation, through its effects on MCs [187].

The current unmet need in the use of intra-articular injections of HA is the duration of the therapeutic effect, which is linked to the time of degradation of HA. The intra-articular inflammatory niche accelerates this process. The number of MCs dramatically increases and the release of lytic enzymes by activated MCs contributes to HA degradation in OA joints. Therefore, ADM, by normalizing MCs activity, may contribute to preventing the degradation of HA and prolong the efficacy of exogenous HA. Moreover, it supports the phenotypical switch of MCs, from an hyperactivated to a physiological state, with consequent restoration of their function of heparin secretion. Since heparin is a precursor of HA, ADM displayed a visco-inductive effect in OA joints in preclinical models [277].

#### 5.2.2. Clinical Data

Intra-articular administration of ADM-HA 2%/1% produced a significant improvement in analgesia, QoL, and functionality in patients suffering from Kellgren and Lawrence grade II-III OA of the knee. The covariates that significantly influenced the results over time were BMI and the presence of dysmetabolic disorders [277]. A retrospective clinical investigation comparing ADM-HA 2%/1% versus HA alone, showed that at 2-years follow-up, ADM improved all components of the WOMAC scale in the treatment of knee OA, physical function, stiffness, and pain, with an overall better result than HA alone [283].

**Figure 2:**
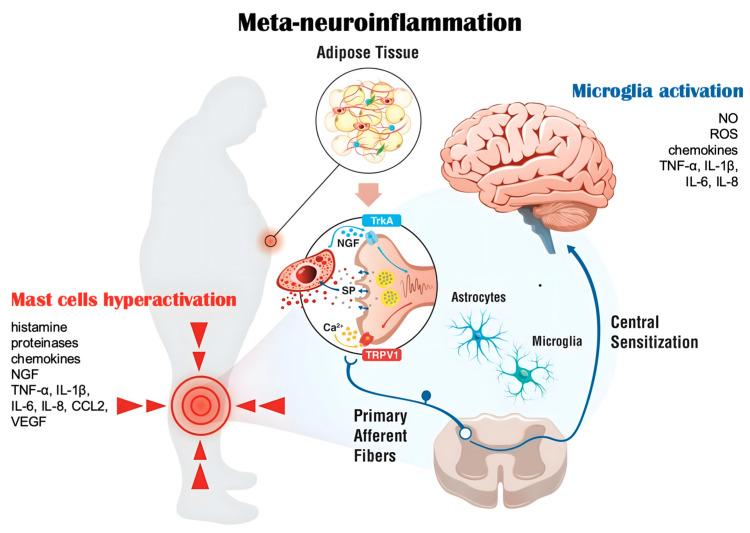
Mechanisms of obesity-related disorders associated with meta-neuroinflammation. Possible targets of treatment for managing obesity-related disorders associated with meta-neuroinflammation include: the pro-inflammatory state sustained by the adipose tissue, the microglial activation in the CNS, and peripheral hyperactivation of mast cells in the peripheral joints. Abbreviations: Ca^2+^, calcium; CCL2, C-C motif ligand 2; IL-1β, Interleukin-1β; IL-6, Interleukin-6; IL-8, Interleukin-8; NGF, Nerve Growth Factor; NO, nitric oxide; ROS, reactive oxygen species; SP, substance P; TNF-α, Tumor Necrosis Factor-α; TrkA, Tropomyosin-Related Kinase A; TRPV1, Transient receptor potential vanilloid 1; VEGF, Vascular Endothelial Growth Factor.

These data suggest the possible combined use in obese patients of ADM-HA, together with other systemic strategies, for targeting meta-neuroinflammation, such as um-PEA and the association m-PEA–rutin and HTyr. Although data on combined effects of these approaches are currently not available, evidence suggests meta-neuroinflammation as an emerging target for optimizing BMI, reducing inflammation biomarkers, and avoiding pain chronification through modulation of primary afferent fibers and prevention of central sensitization (Figure 2).

## 6. Conclusions

Globally, obesity is a serious public health problem associated with increased morbidity and mortality from all causes, including OA, which leads to a reduction in patients’ QoL and life expectancy.

The main findings of this review may be reviewed in the following take-home messages:

- Meta-neuroinflammation is a neologism proposed by authors for describing how the chronic, low-grade systemic inflammation, that occurs in obesity, may trigger oxidative stress and neuroinflammatory processes through the nervous system;
- Obesity could trigger meta-neuroinflammation through dysfunctional adipose tissue, gut dysbiosis and compromised integrity of BBB;
- Meta-neuroinflammation could explain chronic painful diseases, including OA, impaired cognitive function, and mood disorders, observed in obese patients;
- Authors propose a “think outside the box” approach for managing OA in obese patients, by targeting innate immune cells in the brain, mainly microglia, and in periphery, MCs;
- The m-PEA, um-PEA along with its co-micronized formulations, and ADM are promising agents for modulating neuroinflammation in obese patients, with particular benefits for those suffering from OA.

Clinical evidence is still weak, and further studies are warranted to support this hypothesis and open new perspectives for the future of obese patients suffering from painful OA.

## Data Availability

No new data were created or analyzed in this study. Data sharing is not applicable to this article.

## Abbreviations

The following abbreviations are used in this manuscript:

AD	Alzheimer’s disease
ADAMTS	Disintegrin and Metalloproteinase with Thrombospondin Motifs
ADM	Adelmidrol
AGEs	Advanced glycation end-products
AT	Adipose tissue
Aβ	Amyloidbeta-protein
BAT	Beige adipose tissue
BBB	Blood–brain barrier
BDNF	Brain-Derived Neurotrophic Factor
BM	Basement membrane
BMI	Body Mass Index
CB	Cannabinoid Receptors
CCL	C-C motif ligand
CFA	Complete Freund’s Adjuvant
CKD	Chronic kidney disease
CNS	Central nervous system
CVDs	Cardiovascular diseases
DALYs	Disability-Adjusted Life-Years
DM	Diabetes mellitus
DMM	Medial meniscus
DRG	Dorsal root ganglia
ECM	Extracellular matrix
ECMGC	Glycated extracellular matrix
FAAH	Fatty Acid Amide Hydrolase
GM-CSF	Granulocyte-Macrophage Colony-Stimulating Factor
GPx	Glutathione Peroxidase
GSH	Glutathione
GSK-3β	Glycogen synthase kinase-3β
HA	Hyaluronic acid
HIF-1	Hypoxia-Inducible Factor 1
HO-1	Heme Oxygenase-1
HTyr	Hydroxytyrosol
ICAM-1	Intercellular Adhesion Molecule-1
IKK	Inhibitor of Kappa β Kinase
IL	Interleukin
JAM-A	Junctional Adhesion Molecule-A
LBP	low back pain
m-PEA	Micronized palmitoylethanolamide
M1	Pro-inflammatory macrophages
M2	Anti-inflammatory macrophages
MCs	Mast cells
MHC	Major Histocompatibility Complex
MIA	Monosodium iodoacetate
MMPs	Matrix metalloproteinases
m-PEA–rutin	Comicronized palmitoylethanolamide with rutin
mTOR	Mechanistic Target of Rapamycin
NADPH	Nicotinamide Adenine Dinucleotide Phosphate
NAE	N-acylethanolamine
NF-κβ	Nuclear Factor kappa β
NGF	Nerve Growth Factor
NLRP3	NLR Family Pyrin Domain Containing 3
NO	Nitric oxide
NOS	Nitric oxide synthase
NQO1	NAD(P)H Oxidoreductase 1
Nrf	Nuclear Factor Erythroid 2-related Factor
OA	Osteoarthritis
PD	Parkinson’s disease
PEA	Palmitoylethanolamide
PECAM	Platelet Endothelial Cell Adhesion Molecule
PGC-1α	Peroxisome Proliferative Activated Receptor-γ Coactivator-1α
PK	Prokineticin
PKC	Protein Kinase C
PPAR	Peroxisome Proliferator-Activated Receptor
PSGL-1	P-selectin glycoprotein ligand-1
pTau	Hyperphosphorylated Tau
QoL	Quality of Life
RAGE	Advanced glycation end-products receptor
RANK	Receptor Activator of Nuclear Factor-kappa β
C	Rho-kinase
ROS	Reactive Oxygen Species
SCFAs	Short-Chain Fatty Acids
SERCA	Sarcoplasmic Reticulum Calcium ATPase
Sesn2	Sestrin2
SO	Sarcopenic obesity
SOD	Superoxide Dismutase
TGF-β	Transforming Growth Factor-β
TNF-α	Tumor Necrosis Factor-α
TRK	Tropomyosin-Related Kinase
TRPV1	Transient receptor potential vanilloid 1
TWEAK	Tumour Necrosis Factor-Like Weak Inducer of Apoptosis
um-PEA	Ultramicronized palmitoylethanolamide
VEGF	Vascular Endothelial Growth Factor
WAT	White adipose tissue
